# Functional physical training improves fitness and cognitive development in 4~5 years old children

**DOI:** 10.3389/fpsyg.2023.1266216

**Published:** 2023-11-15

**Authors:** Lei Hao, Yongzhao Fan, Xiaojuan Zhang, Xiangjiang Rong, Youping Sun, Kun Liu

**Affiliations:** ^1^College of Physical Education and Health, East China Normal University, Shanghai, China; ^2^Department of Physical Education, Henan Normal University, Xinxiang, Henan, China; ^3^Graduate School, Capital University of Physical Education and Sports, Beijing, China; ^4^Department of Psychiatry, Yale School of Medicine, New Haven, CT, United States; ^5^Brain Peace Science Foundation, New Haven, CT, United States

**Keywords:** functional physical training, fitness development, cognitive development, preschool children, physical and cognitive abilities

## Abstract

**Background:**

Development of physical and cognitive function is very critical in 4~5 years children. It has been addressed in this research if the 18 weeks of specific functional training with or without cognitive training can be effective on improving fitness and cognitive development in 4~5 years preschool children.

**Methods:**

A total of 126 preschool children in the 4~5 age range were selected as participants and randomly assigned to one of four groups: the control group (C), the functional physical training group (P), the cognitive training group (CT), and the functional physical training combined with cognitive training group (PCT).

**Results:**

The results revealed significant pre/post differences in body height and weight among all four groups of children. Furthermore, there was no significant difference in physical fitness between the C group and the CT group after the intervention. However, the children in the P group and the PCT group showed significant improvements in three indicators including standing long jump, continuous jump, and 10-meter shuttle running. Additionally, the children in P group, CT group, and the PCT group demonstrated significant improvement in simple reaction time, attention, and spatial memory. No significant cognitive improvement was found in C group.

**Conclusion:**

Functional physical training with or without cognitive intervention can promote both physical fitness and cognitive development in children aged 4~5 years. Cognitive training alone can significantly improve cognitive function but not physical. Therefore, functional physical training can be used alone to improve the physical and cognitive abilities for aged 4~5 years old children.

## 1. Introduction

Childhood is a critical period for the rapid development of physical fitness and cognitive function (Westfall et al., 2018; Wick et al., 2021). Physical fitness serves as an important indicator of health and has been positively linked to cardiovascular health, fitness levels, cognitive function, mental well-being, and academic achievement (Ortega et al., 2008; Chu et al., 2019; Wassenaar et al., 2019). Additionally, studies have shown a positive correlation between cognitive function and academic performance, as well as overall task performance in children (Fonteyne et al., 2017; Luong et al., 2017). Moreover, this period of childhood is characterized by high behavioral plasticity and sensitivity in both physical and brain development (Pascual-Leone et al., 2005; Lenroot et al., 2009; Chaddock et al., 2012). Thus, it is crucial to implement effective measures to promote physical health and cognitive development in preschool children.

Previous research has implied that excess weighted preschool children, who lack physical exercise, have deficient executive function (Likhitweerawong et al., 2022). The association between physical activity and executive functions also has been found in low-income South African preschool children (Cook et al., 2019). Furthermore, a meta-analysis also suggested that chronic physical exercise could be a promising way to promote multiple aspects of cognitive function, including executive function (Song et al., 2023). The different effectiveness of different intervention on cognitive function has been fully investigated (Diamond and Ling, 2020). Although the previous research suggested that the chronic physical exercise can improve executive functions, some recent systematic reviews argued that the causal relation between chronic exercise training interventions and children’s cognitive functioning is not well established (Diamond and Ling, 2016; Hillman et al., 2018).

For exploring the effect of more differentiated physical exercise on executive function, a specifically designed functional physical exercise, which focuses on enhancing lower body strength, agility, balance, and stability with physical movement games and aerobic exercise volume, had been used in this study in preschool children.

Physical activity has been shown to have numerous benefits for both physical and cognitive development in children (Tucker, 2008). Conversely, insufficient physical activity can lead to weight gain and a decline in cognitive function among children (Ren et al., 2017; Anderson and Durstine, 2019; Fang et al., 2020). However, existing research in this area has primarily focused on school-aged children, with less emphasis on preschool-aged children (Fan and Cao, 2017). The limited research available indicates that preschoolers, including those in China, also experience low levels of physical activity (Barbosa and Oliveira, 2016). Two studies support this finding, with one study revealing that only 35.3% of children in Shanghai met the recommended levels of physical activity, and the other study indicating that only 20–35 min were allocated for physical activities in Hong Kong (Chung et al., 2019; Quan et al., 2019). These time allocations fall far below the minimum recommendations set by the World Health Organization (WHO; Ansari, 2019). Additionally, studies have demonstrated that physical activity patterns in preschool children tend to persist into later childhood (Hardie et al., 2017). Furthermore, emerging evidence suggests that early childhood physical activity is associated with improved cognitive outcomes in later life (Gao et al., 2019a; Daimiel et al., 2020; Lidegaard et al., 2020; Yoong et al., 2020). Therefore, there is a pressing need for physical interventions specifically designed for preschoolers.

Functional physical training is a contemporary training approach that encompasses specialized movement training systems involving incremental, multi-joint, multi-planar, and proprioceptive movements performed under specific load and speed conditions (Zhixiong, 2017). Originally developed for competitive sports, this training method has gained popularity among schoolchildren due to its advanced training concept and engaging techniques (Zhixiong et al., 2018). Additionally, research has indicated the presence of a bidirectional relationship between motor abilities and cognitive skills during early childhood (Nan et al., 2017). Cognitive training has been shown to enhance cognitive function development in school-aged children (Wexler et al., 2016). However, the impact of this physical/cognitive combined training program on the development of physical and cognitive function in preschool children has not been extensively investigated yet. To investigate the synchronized effect of physical and cognitive training on pre-school children development, this study also incorporated a cognitive training component with functional training.

In summary, the physical fitness and cognitive development of preschool children have garnered significant attention, yet there is a lack of structured physical activities aimed at fostering their development. Consequently, this research aims to investigate the impact of functional physical training with or without cognitive training on the physical fitness and cognitive function of preschoolers. The findings of this study will provide valuable insights for enhancing appropriate development for preschool children.

## 2. Materials and methods

### 2.1. Study design and participants

A total of 126 preschool children aged 4 to 5 years (mean age 4.26 ± 0.41, girls: 50%) were selected from various kindergartens in Xicheng District, Beijing, for this research. The kindergartens were randomly selected with coordination by the Xicheng District Education Administration. All the children were randomly divided into four groups: control group (C, *n* = 32), functional physical training group (P, *n* = 31), cognitive training group (CT, *n* = 32), and functional physical training combined with cognitive training intervention group (PCT, *n* = 31). The children in C group did not receive any intervention but participated in regular kindergarten activities. The other three groups underwent an 18-week intervention consisting of functional physical training (40 min), cognitive training (40 min), and a combination of functional physical training (20 min) with cognitive training (20 min), with each training session lasting 40 min, three times per week. The physical training, cognitive training or physical together with cognitive training were performed in outdoor exercise time sections and recess time sections according to the curriculum of the kindergartens. The group-based intervention of the functional physical training and the cognitive training were performed in the indoor gym in the kindergartens by 4 well-trained graduate instructors together with 8 pre-trained preschool teachers. All the instructors and the teachers are female. Each group of preschoolers had one instructor and two teachers in charge of the training to get full engagement.

## 3. Research methods

### 3.1. Morphological and physical fitness tests

The physical fitness assessment in this study was primarily based on the Chinese National Student Physical Fitness Standard for preschool-age children. The assessment consisted of two main parts: morphological tests and physical fitness tests, which included the following prescribed tests: standing long jump (explosive power), sit and reach (flexibility), continuous hop (lower limb strength and coordination), 10-meter shuttle run (agility), balance beam walking (balance), and tennis throw (upper limb and abdominal strength; The State General Administration of Sports, 2003; Liu et al., 2018). The testers involved in the assessment were well-trained and equipped with the necessary knowledge and skills for conducting physical fitness tests. Additionally, all testing instruments were calibrated to ensure accuracy and consistency. Brief descriptions of each test method are provided below.

1. Morphological tests included in this study focused on measuring height and weight. Height and weight were assessed separately using scales with an accuracy of 0.1 cm. These tests provide important morphometric data that contribute to understanding the physical characteristics of the preschool children participating in the study.
2. Physical fitness tests consisted of the following assessments:

Standing long jump: The distance between the starting line and the nearest heel was measured in a straight line.
Sit and reach: The subject extended their arms forward and pushed a cursor as far as possible along a measuring scale, and the maximum value achieved was recorded.
Continuous hop: The test measured the time it took for the subject to complete 10 consecutive hops over soft squares.
10-meter shuttle run: The test measured the shortest time it took for the subject to run a 10-meter distance and return to the starting point.
Balance beam walking: The test measured the shortest time it took for the subject to walk across a 3-meter balance beam.
Tennis throw: The valid score was measured as the straight-line distance between the throwing line and the point where the tennis ball landed.

Each of the physical fitness test measurements was conducted twice, and the results were recorded to one decimal point. These tests provided objective data on the physical capabilities and performance of preschool children.

### 3.2. Cognitive task test

Three cognitive tasks, namely simple reaction time, attention, and spatial location memory breadth, were chosen to evaluate the impact of the intervention program on the cognitive development of preschoolers (Meiling, 2020). These tasks were specifically selected to measure different aspects of cognitive function and provide insights into the cognitive improvements resulting from the intervention. By assessing the performance of preschool children in these tasks before and after the intervention, the study aimed to determine the effects of the program on their cognitive development.

1. Simple reaction time test: This test aims to assess the rapid response ability of children to a fixed and singular visual stimulus. When a green circle appears in the center of the screen, participants are required to press the green button as quickly as possible in response. The test was conducted in groups of 5, with a total of 30 trials using the Psykey Psychometric Test System from Beijing Mind Ark Technology Co. If there was an early button press, the test result would be considered invalid, and the computer would emit a warning tone. The mean value of the valid results was calculated as the simple reaction time.
2. Attention test: The Schulte Table test was utilized primarily to evaluate attentional focus and cognitive stability. This test involves a 3 × 3 square grid with randomly arranged numbers from 1 to 9. Participants are instructed to arrange the numbers in ascending order (1, 2, 3, 4, ... 9) as quickly as possible, aiming for a shorter completion time. If the order of the clicks is incorrect, an auditory tone is triggered until the participant selects the correct order. The test was repeated three times consecutively, and the average value of the valid results was calculated as the attention time.
3. Spatial position memory test: During the test, a 5 × 3 square grid is presented on the computer screen, and the animal will successively show its head from the hole in the ground and then retract again. The subject is asked to look carefully and remember the position and order of the animals. A message will appear at the bottom of the screen, asking the subject to click on the squares in the order in which the animals appear. When the number of animals showing their heads is the same as the number of animals that just appeared, you can click the “OK” button to enter. After 3 attempts of a certain breadth, if not all of them are wrong, the breadth will be increased by 1 and continued until 3 consecutive errors or 12 tasks of a certain breadth are completed.

### 3.3. Functional physical training intervention

The physical fitness intervention for preschool children was developed based on principles of functional physical training and tailored to the characteristics of preschoolers by incorporating gamification elements. The goals of our functional physical training are increasing strength, agility, balance and stability of lower limbs with different incremental, multi-joint, multi-planar, and proprioceptive movements. Furthermore, we designed the fun games gradually using the different difficulty levels of all the movements and performed the physical intervention under specific load and speed conditions. To enhance the enjoyment, novelty, and challenge of the exercises, small and portable equipment was utilized. The intervention lasted for 18 weeks, aiming to maintain appropriate exercise intensity and density for young children while considering their physiological characteristics.

To monitor the heart rate during exercise, a Likang PC-608 finger pulse oximeter was used, with the target heart rate set at 120 to 140 beats per minute (Hengchan et al., 2014). It is important to note that the heart rate was not continuously monitored throughout the exercise to ensure the effectiveness of the training. The main training contents and methods are outlined in Table 1.

**Table 1:** Training content and methods.

Training time (week)	Training content	Methods (Examples)
1 ~ 4	Strength and core stability	Bugs Bunny straight legs jumping, Little horse crossing the river (Obstacle Jump), Winnie the Pooh crawling (hand walk), Two-handed food throwing to fish
5 ~ 6	Balance and core stability	Crossing the Flaming Mountain (Durian ball walking), Bugs Bunny single-leg jump, Crocodile climbing, Crab crawling
7 ~ 8	Speed and aerobic capacity	Bugs Bunny carrying radish, Relay Run, Backward Running
9 ~ 10	Reactivity and flexibility	Multi-directional movement, GO/NO GO, Listen to the password - walk and run alternately, Knee Hug, leg Cradle
11 ~ 18	Functional and cognitive training	Comprehensive Exercises (Combination of the above training methods and cognitive games)

### 3.4. Cognitive training intervention

The cognitive games included in the intervention program comprise four different games (Wexler et al., 2016). Each game has a duration of 20 min. After each game session, the software automatically saves the game progress in the background and adjusts the subsequent cognitive games based on each child’s individual performance.

Game 1: In this game, children track a moving light and click on it when it transforms into a red gem. If the response is correct, the light speeds up, while errors result in a slower pace. As the game progresses, blue gems appear, which should not be clicked. Eventually, the target randomly switches between red and blue gems. To complete the game, children need to identify and click only on the gems that match the previous ones, creating complete gem sequences. Game 2: In this game, pirates throw various objects into the air from a box, and children must click on items belonging to a specific category before they disappear from the screen. Correct responses lead to faster-moving objects, and as the game advances, the difficulty increases by adding six flying objects simultaneously. Game 3: In this game, children are presented with three objects in a row and must choose the correct object from a set of three options to complete the pattern. Game 4: In this game, children are challenged to remember the order in which a group of pirates sitting on a beach raise their hands. The objective is to recall and reproduce the correct sequence of hand raises. The game starts with two pirates, and the level of difficulty increases with successful completions while decreasing with mistakes.

### 3.5. Statistical analysis

The data were reported as the mean ± STDEV and analyzed using SPSS 22.0 software. Initially, normal distribution and homogeneity of variance tests were conducted on the data. The data of every group follow the normal distribution and satisfy the homogeneity of variance. Subsequently, a two-way analysis of variance (ANOVA) with repeated measures was performed to analyze the effects of the intervention and time on the groups. Post-hoc multiple comparisons among the different groups were carried out using the least-significant difference (LSD) test. A significance level of *p* < 0.05 was considered statistically significant.

## 4. Results

### 4.1. Morphological indices of the children

**Figure 1:**
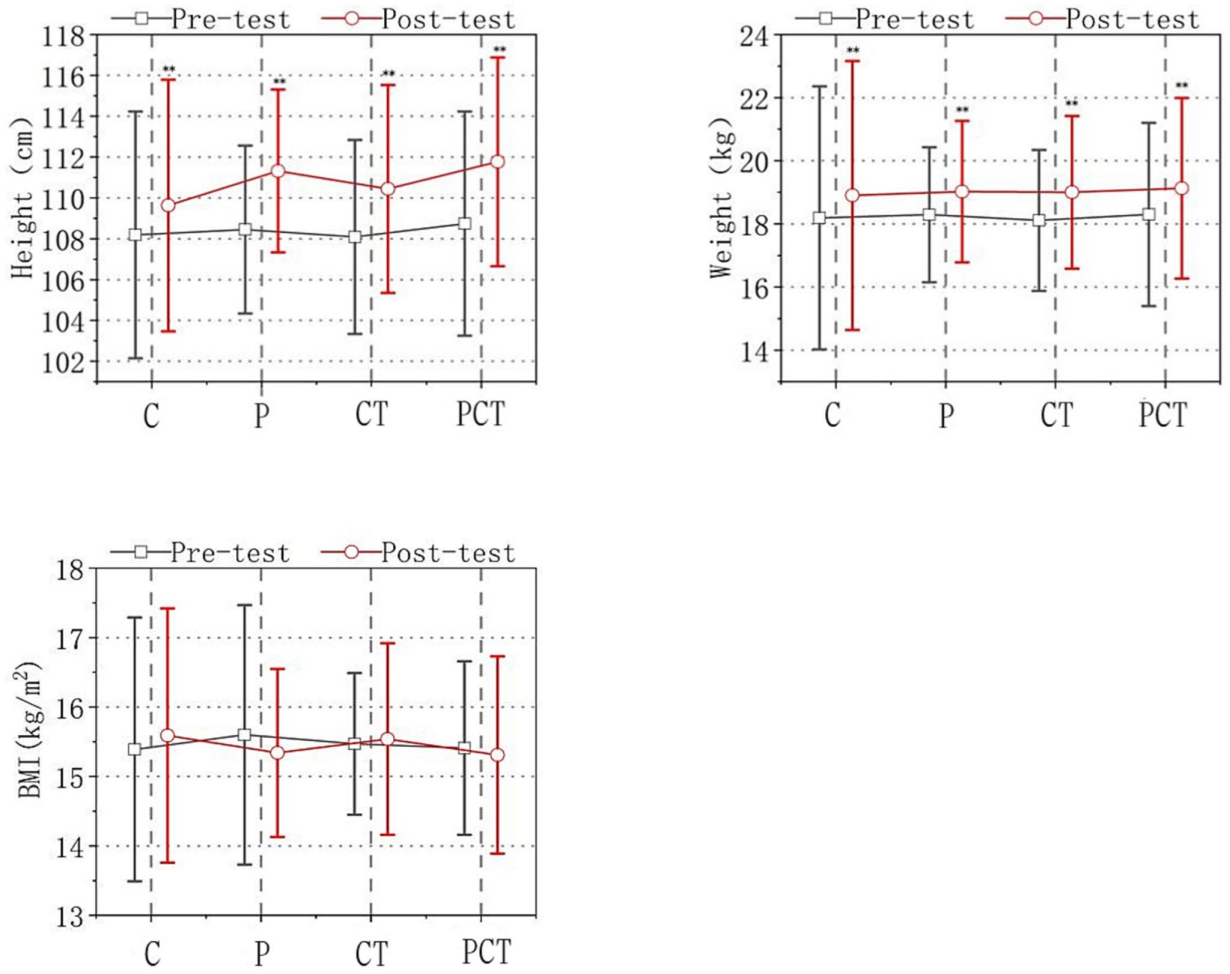
Morphological indices. Note: The four groups were designated as follows: C (control group), P (functional physical training group), CT (cognitive training group), and PCT (functional physical training combined with cognitive training group). Significance levels were indicated as **p < 0.01, denoting a statistically significant difference compared to the pre-test and post-test results in all four groups.

The body heights of the children were 108.9 ± 5.98 cm in C group, 108.35 ± 4.12 cm in P group, 108.00 ± 4.79 cm in CT group, and 108.72 ± 5.53 cm in PCT group at baseline (pre-test). The body height had increased to 109.61 ± 6.02 cm in C group, 109.96 ± 3.93 cm in P group, 109.08 ± 5.29 cm in CT group, and 109.69 ± 5.46 in PCT group after 18 weeks of intervention (post-test). The body weight of the children were 18.19 ± 4.17 kg in C group, 18.29 ± 2.14 kg in P group, 18.11 ± 2.23 kg in CT group, and 18.30 ± 2.90 in PCT group at the baseline. The body weight has increased to 18.66 ± 4.21 kg in C group, 18.96 ± 2.24 kg in P group, 18.87 ± 2.47 kg in CT group and 18.93 ± 2.91 kg in PCT group after 18 weeks of intervention. The BMI of the children were 15.39 ± 1.90 in C group, 15.60 ± 1.87 in P group, 15.47 ± 1.02 in CT group and 15.41 ± 1.25 in PCT group at the baseline and there was no significant change after 18 weeks intervention. All the data above has been shown in Figure 1.

As depicted in Figure 1, the time factor had a significant effect on height and weight [height: *F*(1, 122) = 61.637, *p* < 0.01; weight: F(1, 122) = 242.274, *p* < 0.01], but not on BMI [F(1, 122) = 0.075, *p* > 0.05]. There were no group effects observed for height, weight, and BMI. Additionally, there were no significant differences in height, weight, and BMI between the groups before and after the intervention [height: *F*(3, 122) = 0.493, *p* > 0.05; weight: F(3, 122) = 0.025, *p* > 0.05; BMI: F(3, 122) = 0.065, *p* > 0.05]. Moreover, no interaction effect was found between time and group [height: F(3, 122) = 0.257, *p* > 0.05; weight: F(3, 122) = 0.58, *p* > 0.05; BMI: F(3, 122) = 0.283, *p* > 0.05].

### 4.2. The result of the physical fitness test

**Figure 2:**
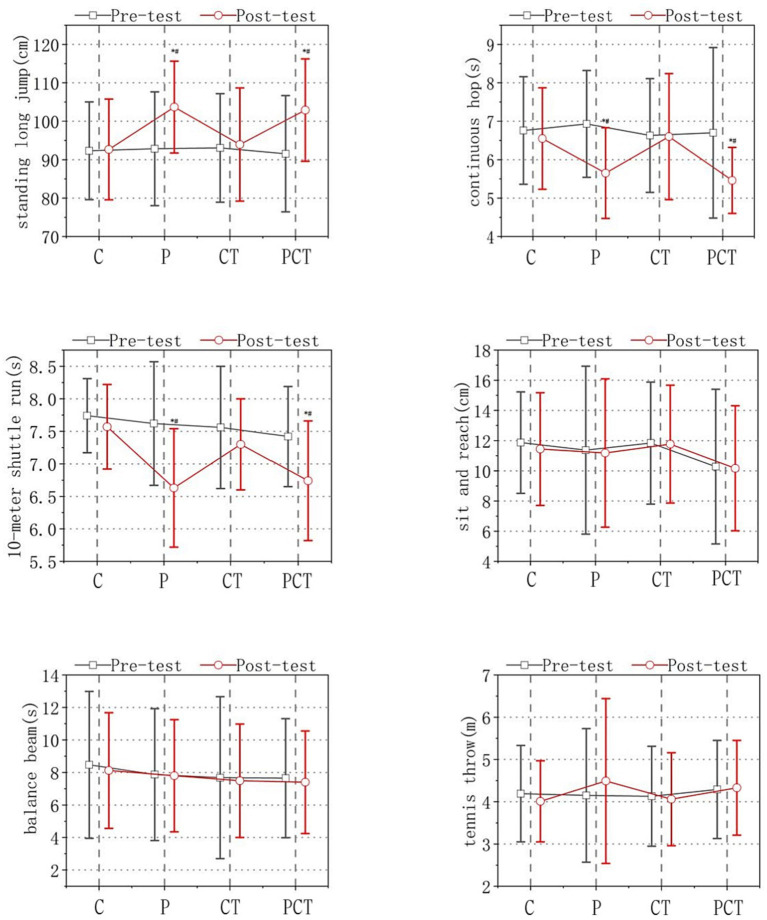
Results of physical fitness test. Note: The four groups were designated as follows: C (control group), P (functional physical training group), CT (cognitive training group), and PCT (functional physical training combined with cognitive training group). Significance levels were indicated as *p < 0.05, denoting a statistically significant difference compared to the pre-test and post-test results in all four groups.

The standing long jump scores were 92.31 ± 12.71 cm in C group, 92.84 ± 14.79 cm in P group, 93.06 ± 14.12 cm in CT group and 91.94 ± 15.13 cm in PCT group at baseline (pre-test). The standing long jump scores were 92.66 ± 13.10 cm in C group and 93.64 ± 14.73 cm in CT group without significant changes, and had improved to 103.69 ± 11.59 cm in P group and 103.56 ± 16.41 in PCT group after 18 weeks intervention (post-test). The continuous hop scores were 6.76 ± 1.40 s (s) in C group, 6.93 ± 1.39 s in P group, 6.63 ± 1.48 s in CT group and 6.70 ± 2.22 s in PCT group at baseline (pre-test). The continuous hop scores were at 6.58 ± 0.76 s in C group and 6.60 ± 1.64 s in CT group without significant change, and decreased to 5.65 ± 1.18 s in P group and 5.46 ± 0.86 s in PCT group after 18 weeks of intervention (post-test). The 10-meter shuttle run of the children were 7.74 ± 0.57 s in C group, 7.62 ± 0.95 s in P group, 7.56 ± 0.94 s in CT group and 7.42 ± 0.77 s in PCT group at the baseline (pre-test). The 10-meter shuttle run scores has decreased to 7.54 ± 0.57 s in C group and 7.33 ± 0.70 s in CT group without significant change, and decreased significantly to 6.63 ± 0.91 s in P group and 6.74 ± 0.92 s in PCT group after 18 weeks of intervention (post-test). The sit and reach scores of the children were 11.87 ± 3.36 cm in C group, 11.37 ± 5.56 cm in P group, 11.84 ± 4.04 cm in CT group, and 10.32 ± 5.12 cm in PCT group at baseline (pre-test). The sit and reach scores were 11.44 ± 4.39 cm in C group, 11.05 ± 5.23 cm in P group, 11.70 ± 3.99 cm in CT group, and 10.17 ± 4.14 cm in PCT group after 18 weeks of intervention (post-test). There was no significant change after the intervention. The balance beam walking scores of the children were 8.47 ± 4.52 s in C group, 7.87 ± 4.06 s in P group, 7.76 ± 4.99 s in CT group, and 7.65 ± 3.66 s in PCT group at the baseline (pre-test). The balance beam walking scores of the children were 8.12 ± 3.56 s in C group, 7.80 ± 3.45 s in P group, 7.49 ± 3.49 s in CT group and 7.40 ± 3.15 s in PCT group after 18 weeks of intervention (post-test). There was no significant change after the intervention. The tennis throw scores of the children were 4.19 ± 1.14 m in C group, 4.15 ± 1.58 m in P group, 4.13 ± 1.18 m in CT group, and 4.29 ± 1.16 m in PCT group at baseline (pre-test). The tennis throw scores were 4.01 ± 0.96 m in C group, 4.49 ± 1.95 m in P group, 4.06 ± 1.10 m in CT group, and 4.33 ± 1.12 m in PCT group after 18 weeks of intervention (post-test). There was no significant change after the intervention. All the data above has been shown in Figure 2.
As shown in Figure 2, the time effects were found to have a significant impact on the standing long jump, continuous hop, and 10-meter shuttle run [standing long jump: *F*(1, 122) = 42.257, *p* < 0.05; continuous hop: F(1, 122) = 20.26, *p* < 0.05; 10-meter shuttle run: F(1, 122) = 28.986, *p* < 0.05]. However, there were no significant time effects observed for the sit and reach, balance beam, and tennis throw tests [sit and reach: F(1, 122) = 0.483, *p* > 0.05; balance beam: F(1, 122) = 0.365, *p* > 0.05; tennis throw: F(1, 122) = 0.08, *p* > 0.05] in the P and PCT groups. Similar effects were observed for the grouping effect [standing long jump: *F*(3, 122) = 12.871, *p* < 0.05; continuous hop: F(3, 122) = 1.61, *p* > 0.05; 10-meter shuttle run: F(3, 122) = 6.638, *p* < 0.05; sit and reach: F(3, 122) = 0.94, *p* > 0.05; balance beam: F(3, 122) = 0.345, *p* > 0.05; tennis throw: F(3, 122) = 0.38, *p* > 0.05].
A significant interaction effect between time and group was observed for the standing long jump, continuous hop, and 10-meter shuttle run tests [standing long jump: F(3, 122) = 12.871, *p* < 0.05; continuous hop: F(3, 122) = 4.761, *p* < 0.05; 10-meter shuttle run: F(3, 122) = 11.035, *p* < 0.05]. However, there were no significant interaction effects observed for the sit and reach, balance beam, and tennis throw tests [sit and reach: F(3, 122) = 0.081, *p* > 0.05; balance beam: F(3, 122) = 0.106, *p* > 0.05; tennis throw: F(3, 122) = 0.944, *p* > 0.05].

### 4.3. The result of the cognitive test

**Figure 3:**
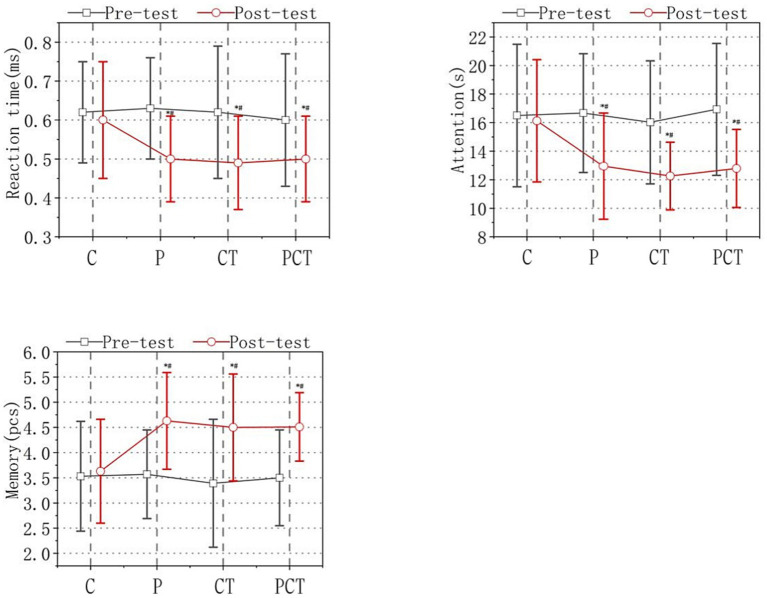
Results of cognitive test. Note: the four groups were designated as follows: C (control group), P (functional physical training group), CT (cognitive training group), and PCT (functional physical training combined with cognitive training group). Significance levels were indicated as *p < 0.05, denoting a statistically significant difference compared to the pre-test and post-test results in all four groups.

The reaction time scores were 0.62 ± 0.13 s (s) in C group, 0.63 ± 0.13 s in P group, 0.62 ± 0.17 s in CT group and 0.60 ± 0.17 s in PCT group at baseline (pre-test). The reaction time score had no significant change to 0.60 ± 0.15 s in C group, and had improved to 0.50 ± 0.12 s in P group, 0.49 ± 0.12 s in CT group and 0.51 ± 0.15 s in PCT group after 18 weeks intervention (post-test). The attention scores were 16.50 ± 4.99 s in C group, 16.67 ± 4.16 s in P group, 16.02 ± 4.31 s in CT group and 16.93 ± 4.04 s in PCT group at the baseline (pre-test). The attention score had no significant change at 16.13 ± 4.28 s in C group, and had decreased to 13.66 ± 4.76 s in P group, 12.55 ± 5.08 s in CT group and 12.92 ± 4.04 in PCT group after 18 weeks of intervention (post-test). The memory test scores were 3.67 ± 1.30 in C group, 3.88 ± 1.10 in P group, 3.67 ± 1.59 in CT group and 3.59 ± 1.26 in PCT group at the baseline (pre-test). The memory score had no significant change at 3.56 ± 1.12 in C group, and had improved to 4.59 ± 1.31 in P group, 4.50 ± 1.47 in CT group and 4.50 ± 1.12 in PCT group after 18 weeks of intervention (post-test). All the data above has been shown in Figure 3.

As shown in Figure 3, the time effects had a significant impact on reaction time, attention, and memory [reaction time: *F*(1, 122) = 39.781, *p* < 0.05; attention: F(1, 122) = 51.761, *p* < 0.05; memory: F(1, 122) = 248.129, *p* < 0.05]. The group effect also had a significant impact [reaction time: *F*(3, 122) = 2.068, *p* < 0.05; attention: F(3, 122) = 2.568, *p* > 0.05; memory: F(3, 122) = 1.792, *p* > 0.05]. A significant interaction effect was observed between time and group in reaction time, attention, and memory tests [reaction time: F(3, 122) = 2.529, *p* > 0.05; attention: F(3, 122) = 4.511, *p* < 0.05; memory: F(3, 122) = 22.408, *p* < 0.05].

## 5. Discussion

Childhood is a critical period characterized by significant physiological, structural, and functional changes (Teicher et al., 2016; Gilmore et al., 2018). Physical fitness plays a crucial role in child development and is considered a predictor of health outcomes. The physical fitness has been found to have a positive association with cognitive function and brain health (Esteban-Cornejo et al., 2017; Haga et al., 2019). While physical fitness is influenced by various factors, it is particularly influenced by physical activity habits (Daimiel et al., 2020). Insufficient physical activity has been linked to negative effects on the healthy development of preschool children (Gao et al., 2019b). On the other hand, well-designed physical activity interventions have been shown to significantly promote both physical and cognitive development in school-age children (Mead et al., 2017). Therefore, implementing appropriate intervention is essential to promote the healthy development of preschool children.

The initial measurements of height and weight in the four groups showed no significant differences, indicating that the groups were initially comparable. After the 18-week intervention, there was a significant increase in both height and weight across all four groups of children. These findings are consistent with previous studies that have also observed significant changes in height and weight following interventions (Eliakim et al., 2007). It is worth noting that the age range of 5–6 years corresponds to a rapid stage of growth and development in children, which further supports the observed increases in height and weight (WHO Multicentre Growth Reference Study Group and de Onis, 2006).

However, there was a notable difference in the results of this study compared to a previous experiment. Specifically in the previous experiment, the weight of children in the experimental group was significantly lower than that of the control group (Bocca et al., 2012). This discrepancy can be attributed to the implementation of a dietary intervention in the study, which effectively reduced body fat levels in children. It is likely that the dietary intervention played a significant role in influencing the weight outcomes and accounts for the different results between the two studies.

Physical fitness encompasses various aspects of the human body, including its morphological structure, physiological functions, and psychological factors. It is influenced by both genetic factors and acquired characteristics (Roy et al., 2010). Physical fitness is closely associated with numerous health benefits and behavioral outcomes (Bouchard et al., 2012). Certain components of physical fitness have been identified as particularly important indicators of various health outcomes in young individuals (Ortega et al., 2008). In this study, advanced functional physical training was employed to promote the development of physical fitness in preschool children. The results indicated that the standing long jump, continuous hop, and 10-meter shuttle run performance of the P group and the PCT group were significantly better than those of the C group and the CT group. This suggests that the 18-week functional physical training can effectively enhance explosive force, lower limb muscle strength, coordination, and agility in preschoolers. These findings are consistent with similar studies that have demonstrated significant improvements in body composition after more than 6 weeks of physical activity interventions (Puder et al., 2011; Foulkes et al., 2017). Moreover, changes in cardiorespiratory fitness, lower limb muscle strength, and speed agility have been found to be associated with changes in body composition in preschool children (García-Hermoso et al., 2020).

The experimental groups in this study engaged in regular moderate-to-vigorous physical activity, which has been shown to be positively correlated with muscle strength, explosive power, balance, agility, and aerobic fitness in preschoolers (Fang et al., 2017). Additionally, numerous studies have established a positive correlation between physical fitness and motor ability in children (Aadland et al., 2017; Nan et al., 2017). One study specifically confirmed that functional physical training, as a novel form of training, is highly beneficial in improving the fundamental motor skills of preschool children (Zhexiao et al., 2016). Therefore, the results of this study are reliable and in line with existing evidence.

Cognitive function encompasses various sub-functions such as response, attention, memory, learning, language, perception, and executive function (Fiocco and Yaffe, 2010). Among these, response time is an important indicator for assessing brain function, attention processes, cognitive flexibility, behavior, and performance (Stuss et al., 2005; Hillman et al., 2014; Ángel et al., 2018). The development of attention and working memory skills is closely related to children’s health and behavioral performance (Conklin et al., 2007; Oberauer, 2019). In this study, these indicators were chosen to evaluate the cognitive function of school-age children. The results of the study revealed that simple reaction time, attention, and spatial memory were significantly better in the P group, CT group, and PCT group compared to the C group, with no significant differences observed among the three intervention groups. The cognitive games used in this study were designed based on the principle of neuroplasticity, and previous research has demonstrated that playing such games can significantly improve focused attention, response inhibition, working memory, and cognitive flexibility in school-age children (Klingberg et al., 2005). By engaging in these cognitive games, preschool children in the experiment were able to dynamically reconfigure their neural systems, leading to the observed improvements in cognitive function. Furthermore, childhood is a period characterized by rapid structural and functional development of the central nervous system and brain, and the brain appears to be particularly responsive to exercise during this stage (Schacter et al., 2004; Khan and Hillman, 2014; Hillman and Biggan, 2017). Studies have shown that children with higher aerobic capacity and agility exhibit larger brain volumes in the gray matter, frontal lobes, hippocampus, and caudate nucleus, as well as more efficient neuroelectric processing during cognitive tasks. These differences in brain structures and functions contribute to variations in working memory, cognitive control, and attention among children (Donnelly et al., 2016; Santana et al., 2017). Additionally, physical activity-induced increases in cerebral blood flow and the release of neurotrophic factors may also contribute to the observed changes in cognitive function (Alfini et al., 2019; Tari et al., 2019).

Generally based on our research results, we have demonstrated that the specially designed functional physical training can improve both physical fitness and cognitive function in 4~5 years old preschool children. At the same time, the cognitive training can improve the cognitive function but not the physical fitness. Furthermore, the enhanced cognitive function is possibly induced by more improvement of lower limbs’ function such as jumping, hopping, and shuttle running. Although our previous research (Ortega et al., 2008) had suggested that lower limb strength and balance are correlated with executive functions such as processing speed and attention, it is the first time in this controlled study that we have demonstrated that the functional training aiming at lower limbs strength, agility and rhythm can improve the cognitive functions such as processing speed, attention and spatial memory. The underlying mechanisms might be related to more sensory stimulation induced by better physical activity space and more neurotrophic factors releasing with better physical exercise with lower limbs. However, since the limitation of the research design in this study, such as the physical intervention mostly focused on lower limbs movements, the cognitive assessment mostly focused on executive function, and no cognition correlated biological samples or brain images had been addressed in our research, the more specific underlying mechanisms still need to be further investigated.

## 6. Conclusion

Combining functional physical training and cognitive training has shown to be effective in promoting physical fitness and cognitive development. Cognitive training alone significantly improves cognitive function, while functional physical training significantly enhances both physical fitness and cognitive function in children aged 4~5 years.

## Data availability statement

The raw data supporting the conclusions of this article will be made available by the authors, without undue reservation.

## Ethics statement

The studies involving humans were approved by the institutional ethical committee of the Capital University of Physical Education and Sports, Beijing, China (2017A03). The studies were conducted in accordance with the local legislation and institutional requirements. Written informed consent for participation in this study was provided by the participants’ legal guardians/next of kin.

## Author contributions

LH: Conceptualization, Software, Validation, Visualization, Writing – original draft, Writing – review & editing. YF: Data curation, Formal analysis, Investigation, Methodology, Software, Validation, Visualization, Writing – original draft. XZ: Investigation, Project administration. XR: Project administration. YS: Supervision, Writing – review & editing. KL: Conceptualization, Methodology, Project administration, Resources, Validation, Writing – review & editing.

## References

[ref1] Aadland K. N. Moe V. F. Aadland E. Anderssen S. A. Resaland G. K. Ommundsen Y. (2017). Relationships between physical activity, sedentary time, aerobic fitness, motor skills and executive function and academic performance in children. Ment. Health Phys. Act. 12, 10–18. doi: 10.1016/j.mhpa.2017.01.001

[ref2] Alfini A. J. Weiss L. R. Nielson K. A. Verber M. D. Smith J. C. (2019). Resting cerebral blood flow after exercise training in mild cognitive impairment. J. Alzheimers Dis. 67, 671–684. doi: 10.3233/jad-180728, PMID: 30636734 PMC6444938

[ref3] Anderson E. Durstine J. L. (2019). Physical activity, exercise, and chronic diseases: a brief review. Sports Med. Heal. Sci. 1, 3–10. doi: 10.1016/j.smhs.2019.08.006, PMID: 35782456 PMC9219321

[ref4] Ángel L.-R. P. Robles-Fuentes A. García-Pinillos F. Salas-Sánchez J. (2018). Reaction times of preschool children on the ruler drop test: a cross-sectional study with reference values. Percept. Mot. Skills 125, 866–878. doi: 10.1177/0031512518789563 30032724

[ref5] Ansari M. T. (2019). WHO guidelines on physical activity, sedentary behaviour and sleep for children under 5 years of age. Geneva: World Health Organization. 31091057

[ref6] Barbosa H. C. Oliveira A. R. D. (2016). Physical activity of preschool children: a review. OMICS Int. 1:111. doi: 10.4172/2573-0312.1000111

[ref7] Bocca G. Corpeleijn E. Stolk R. P. Sauer P. J. J. (2012). Results of a multidisciplinary treatment program in 3-year-old to 5-year-old overweight or obese children: a randomized controlled clinical trial. Arch. Pediatr. Adolesc. Med. 166, 1109–1115. doi: 10.1001/archpediatrics.2012.1638 23108941

[ref8] Bouchard C. Blair S. N. Haskell W. L. (2012). Physical activity and health. Human Kinetics. Champaign, Illinois.

[ref9] Chaddock L. Hillman C. H. Pontifex M. B. Johnson C. R. Raine L. B. Kramer A. F. (2012). Childhood aerobic fitness predicts cognitive performance one year later. J. Sports Sci. 30, 421–430. doi: 10.1080/02640414.2011.647706, PMID: 22260155

[ref10] Chu C. H. Chen F. T. Pontifex M. B. Sun Y. Chang Y. K. (2019). Health-related physical fitness, academic achievement, and neuroelectric measures in children and adolescents. Int. J. Sport Exer. Psychol. 17, 117–132. doi: 10.1080/1612197X.2016.1223420

[ref11] Chung J. W. Yee W. Sum W. Yan V. C. (2019). The analysis of changes in the physical fitness of Hong Kong preschoolers following the adoption of an integrated physical fitness curriculum. Int. J. Sci. Healthcare Res. 4, 185–193.

[ref12] Conklin H. M. Luciana M. Hooper C. J. Yarger R. S. (2007). Working memory performance in typically developing children and adolescents: behavioral evidence of protracted frontal lobe development. Dev. Neuropsychol. 31, 103–128. doi: 10.1207/s15326942dn3101_6, PMID: 17305440

[ref13] Cook C. J. Howard S. J. Scerif G. Twine R. Kahn K. Norris S. A.. (2019). Associations of physical activity and gross motor skills with executive function in preschool children from low-income south African settings. Dev. Sci. 22:e12820. doi: 10.1111/desc.12820, PMID: 30801916

[ref14] Daimiel L. Martínez-González M. A. Corella D. Salas-Salvadó J. Schröder H. Vioque J.. (2020). Physical fitness and physical activity association with cognitive function and quality of life: baseline cross-sectional analysis of the PREDIMED-plus trial. Sci. Rep. 10, 3472–3412. doi: 10.1038/s41598-020-59458-6, PMID: 32103064 PMC7044289

[ref15] Diamond A. Ling D. S. (2016). Conclusions about interventions, programs, and approaches for improving executive functions that appear justified and those that, despite much hype, do not. Dev. Cogn. Neurosci. 18, 34–48. doi: 10.1016/j.dcn.2015.11.005, PMID: 26749076 PMC5108631

[ref16] Diamond A. Ling D. S. (2020). “Review of the evidence on, and fundamental questions about, efforts to improve executive functions, including working memory” in Cognitive and working memory training: Perspectives from psychology, neuroscience, and human development. eds. Novick J. M. Bunting M. F. Dougherty M. R. Engle R. W. (Oxford: Oxford University Press), 143–431.

[ref17] Donnelly J. E. Hillman C. Castelli D. Etnier J. L. Szabo-Reed A. (2016). Physical activity, fitness, cognitive function, and academic achievement in children: a systematic review. Med. Sci. Sports Exerc. 48, 1197–1222. doi: 10.1249/MSS.0000000000000901, PMID: 27182986 PMC4874515

[ref18] Eliakim A. Nemet D. Balakirski Y. Epstein Y. (2007). The effects of nutritional-physical activity school-based intervention on fatness and fitness in preschool children. J. pediatric endocrinology & metabolism: JPEM 20, 711–718. doi: 10.1515/JPEM.2007.20.6.711, PMID: 17663296

[ref19] Esteban-Cornejo I. Cadenas-Sánchez C. Contreras-Rodriguez O. Verdejo-Roman J. Mora-González J. Migueles J. H.. (2017). A whole brain volumetric approach in overweight/obese children: examining the association with different physical fitness components and academic performance. The active brains project. Neuroimage 159, 346–354. doi: 10.1016/j.neuroimage.2017.08.011, PMID: 28789992

[ref20] Fan X. Cao Z. B. (2017). Physical activity among Chinese school-aged children: national prevalence estimates from the 2016 physical activity and fitness in China-the youth study. J. Sport Health Sci. 6, 388–394. doi: 10.1016/j.jshs.2017.09.006, PMID: 30356592 PMC6189233

[ref21] Fang H. Minghui Q. Tang Z. Shunli S. Jiayi Z. Hanbin Z.. (2017). Relationship between physical activity and physical fitness in preschool children: a cross-sectional study. Biomed. Res. Int. 2017, 1–8. doi: 10.1155/2017/9314026, PMID: 29359160 PMC5735582

[ref22] Fang C. Zhang J. Zhou T. Li L. Lu Y. Gao Z.. (2020). Associations between daily step counts and physical fitness in preschool children. J. Clin. Med. 9:163. doi: 10.3390/jcm9010163, PMID: 31936133 PMC7019471

[ref23] Fiocco A. J. Yaffe K. (2010). Defining successful aging: the importance of including cognitive function over time. Arch. Neurol. 67, 876–880. doi: 10.1001/archneurol.2010.130, PMID: 20625097

[ref24] Fonteyne L. Duyck W. De Fruyt F. (2017). Program-specific prediction of academic achievement on the basis of cognitive and non-cognitive factors. Learn. Individ. Differ. 56, 34–48. doi: 10.1016/j.lindif.2017.05.003

[ref25] Foulkes J. D. Knowles Z. Fairclough S. J. Stratton G. O’Dwyer M. Ridgers N. D.. (2017). Effect of a 6-week active play intervention on fundamental movement skill competence of preschool children: a cluster randomized controlled trial. Percept. Mot. Skills 124, 393–412. doi: 10.1177/0031512516685200, PMID: 28361654

[ref26] Gao Z. Lee J. E. Zeng N. Pope Z. C. Li X. (2019b). Home-based exergaming on preschoolers’ energy expenditure, cardiovascular fitness, body mass index and cognitive flexibility: a randomized controlled trial. J. Clin. Med. 8:1745. doi: 10.3390/jcm8101745, PMID: 31640158 PMC6832462

[ref27] Gao Z. Zeng N. Pope Z. C. Wang R. Yu F. (2019a). Effects of exergaming on motor skill competence, perceived competence, and physical activity in preschool children. J. Sport Health Sci. 8, 106–113. doi: 10.1016/j.jshs.2018.12.001, PMID: 30997256 PMC6450920

[ref28] García-Hermoso A. Alonso-Martinez A. M. Ramírez-Vélez R. Izquierdo M. (2020). Effects of exercise intervention on health-related physical fitness and blood pressure in preschool children: a systematic review and meta-analysis of randomized controlled trials. Sports Med. 50, 187–203. doi: 10.1007/s40279-019-01191-w, PMID: 31556009

[ref29] Gilmore J. H. Knickmeyer R. C. Gao W. (2018). Imaging structural and functional brain development in early childhood. Nat. Rev. Neurosci. 19, 123–137. doi: 10.1038/nrn.2018.1, PMID: 29449712 PMC5987539

[ref30] Haga M. Haapala E. A. Sigmundsson H. (2019). Physical fitness. The Encyclopedia of Child and Adolescent Development: Wiley-Blackwell, New York, p. 1–10.

[ref31] Hardie M. M. Rowe D. A. Woods C. B. (2017). Impact of physical activity domains on subsequent physical activity in youth: a 5-year longitudinal study. J. Sports Sci. 35, 262–268. doi: 10.1080/02640414.2016.1161219, PMID: 27067829

[ref32] Hengchan Y. Aiguo C. Zheng M. Xingnan L. Min L. (2014). A follow-up study on two kinds of exercise intervention programs for children’s executive function. Sports. Science 34, 24–28. doi: 10.16469/j.css.2014.03.001

[ref33] Hillman C. H. Biggan J. R. (2017). A review of childhood physical activity, brain, and cognition: perspectives on the future. Pediatr. Exerc. Sci. 29, 170–176. doi: 10.1123/pes.2016-0125, PMID: 27615274

[ref34] Hillman C. H. McAuley E. Erickson K. I. Liu-Ambrose T. Kramer A. F. (2018). On mindful and mindless physical activity and executive function: a response to Diamond and Ling (2016). Dev. Cogn. Neurosci. 37:100529. doi: 10.1016/j.dcn.2018.01.006, PMID: 30318345 PMC6969305

[ref35] Hillman C. H. Pontifex M. B. Castelli D. M. Khan N. A. Raine L. B. Scudder M. R.. (2014). Effects of the FITKids randomized controlled trial on executive control and brain function. Pediatrics 134, e1063–e1071. doi: 10.1542/peds.2013-3219 25266425 PMC4179093

[ref36] Khan N. A. Hillman C. H. (2014). The relation of childhood physical activity and aerobic fitness to brain function and cognition: a review. Pediatr. Exerc. Sci. 26, 138–146. doi: 10.1123/pes.2013-0125, PMID: 24722921

[ref37] Klingberg T. Fernell E. Olesen P. J. Johnson M. Gustafsson P. Dahlstrom K.. (2005). Computerized training of working memory in children with ADHD-a randomized, controlled trial. J. Am. Acad. Child Adolesc. Psychiatry 44, 177–186. doi: 10.1097/00004583-200502000-00010, PMID: 15689731

[ref38] Lenroot R. K. Schmitt J. E. Ordaz S. J. Wallace G. L. Neale M. C. Lerch J. P.. (2009). Differences in genetic and environmental influences on the human cerebral cortex associated with development during childhood and adolescence. Hum. Brain Mapp. 30, 163–174. doi: 10.1002/hbm.20494 18041741 PMC6870600

[ref39] Lidegaard M. Lerche A. F. Munch P. K. Schmidt K. G. Rasmussen C. L. Rasmussen C. D. N.. (2020). Can childcare work be designed to promote moderate and vigorous physical activity, cardiorespiratory fitness and health? Study protocol for the goldilocks-childcare randomised controlled trial. BMC Public Health 20, 237–211. doi: 10.1186/s12889-020-8291-y, PMID: 32066404 PMC7026977

[ref40] Likhitweerawong N. Khorana J. Boonchooduang N. Phinyo P. Patumanond J. Louthrenoo O. (2022). Association between executive function and excess weight in pre-school children. PLoS One 17:e0275711. doi: 10.1371/journal.pone.0275711, PMID: 36215258 PMC9550082

[ref41] Liu X. Xiang Z. Liu C. Shi X. Yi X. Cheng M.. (2018). Risk factors associated with poor physical fitness in three- to six-year-old children in tujia-nationality settlement of China. Evid. Based Complement. Alternat. Med. 2018:1–9. doi: 10.1155/2018/5702190, PMID: 30532796 PMC6250012

[ref42] Luong C. Strobel A. Wollschläger R. Greiff S. Vainikainen M.-P. Preckel F. (2017). Need for cognition in children and adolescents: behavioral correlates and relations to academic achievement and potential. Learn. Individ. Differ. 53, 103–113. doi: 10.1016/j.lindif.2016.10.019

[ref43] Mead E. Brown T. Rees K. Azevedo L. B. Ells L. J. (2017). Diet, physical activity and behavioural interventions for the treatment of overweight or obese children from the age of 6 to 11 years. Cochrane Database Syst. Rev. 2017:6. doi: 10.1002/14651858.CD012651, PMID: 28639319 PMC6481885

[ref44] Meiling Z. (2020). The effects of two kinds of training intervention programs on physical fitness and different cognitive tasks of preschool children. J. Beijing University of Sports 43, 89–97. doi: 10.19582/j.cnki.11-3785/g8.2020.05.009

[ref45] Nan Z. Mohammad A. Haichun S. Xu W. Ping X. Zan G. (2017). Effects of physical activity on motor skills and cognitive development in early childhood: a systematic review. Biomed. Res. Int. 2017:1–13. doi: 10.1155/2017/2760716, PMID: 29387718 PMC5745693

[ref46] Oberauer K. (2019). Working memory and attention-a conceptual analysis and review. J. Cogn. 2:36. doi: 10.5334/joc.58, PMID: 31517246 PMC6688548

[ref47] Ortega F. Ruiz J. R. Castillo M. J. Sjöström M. (2008). Physical fitness in childhood and adolescence: a powerful marker of health. Int. J. Obes. 32, 1–11. doi: 10.1038/sj.ijo.0803774, PMID: 18043605

[ref48] Pascual-Leone A. Amedi A. Fregni F. Merabet L. B. (2005). The plastic human brain cortex. Annu. Rev. Neurosci. 28, 377–401. doi: 10.1146/annurev.neuro.27.070203.144216 16022601

[ref49] Puder J. J. Marques-Vidal P. Schindler C. Zahner L. Niederer I. Bürgi F.. (2011). Effect of multidimensional lifestyle intervention on fitness and adiposity in predominantly migrant preschool children (Ballabeina): cluster randomised controlled trial. BMJ 343:d6195. doi: 10.1136/bmj.d6195, PMID: 21998346 PMC3192456

[ref50] Quan M. Zhang H. Zhang J. Zhou T. Zhang J. Zhao G.. (2019). Are preschool children active enough in Shanghai: an accelerometer-based cross-sectional study. BMJ Open 9:e024090. doi: 10.1136/bmjopen-2018-024090, PMID: 31028035 PMC6502006

[ref51] Ren H. Zhou Z. Liu W. K. Wang X. Yin Z. (2017). Excessive homework, inadequate sleep, physical inactivity and screen viewing time are major contributors to high paediatric obesity. Acta Paediatr. 106, 120–127. doi: 10.1111/apa.13640, PMID: 27759894 PMC6680318

[ref52] Roy T. Springer B. A. McNulty V. Butler N. L. (2010). Physical fitness. Mil. Med. 175, 14–20. doi: 10.7205/MILMED-D-10-0005820108837

[ref53] Santana C. C. A. Azevedo L. B. Cattuzzo M. T. Hill J. O. Andrade L. P. Prado W. L. (2017). Physical fitness and academic performance in youth: a systematic review. Scand. J. Med. Sci. Sports 27, 579–603. doi: 10.1111/sms.12773 27714852

[ref54] Schacter D. L. Dobbins I. G. Schnyer D. N. (2004). Specificity of priming: a cognitive neuroscience perspective. Nature Rev. Neurosci. 5, 853–862. doi: 10.1038/nrn1534 15496863

[ref55] Song H. Wang J. J. Zhang B. Shi L. Lau P. W. C. (2023). Do acute and chronic physical activity interventions affect the cognitive function of preschool children? a meta-analysis. Psychol. Sport Exerc. 67:102419. doi: 10.1016/j.psychsport.2023.102419 37665872

[ref56] Stuss D. T. Alexander M. P. Shallice T. Picton T. W. Binns M. A. Macdonald R.. (2005). Multiple frontal systems controlling response speed. Neuropsychologia 43, 396–417. doi: 10.1016/j.neuropsychologia.2004.06.010 15707616

[ref57] Tari A. R. Norevik C. S. Scrimgeour N. R. Kobro-Flatmoen A. Wislff U. (2019). Are the neuroprotective effects of exercise training systemically mediated? Prog. Cardiovasc. Dis. 62, 94–101. doi: 10.1016/j.pcad.2019.02.003 30802460

[ref58] Teicher M. H. Samson J. A. Anderson C. M. Ohashi K. (2016). The effects of childhood maltreatment on brain structure, function and connectivity. Nat. Rev. Neurosci. 17, 652–666. doi: 10.1038/nrn.2016.111 27640984

[ref59] The State General Administration of Sports. (2003). Chinese National Measurement Standards on People's physical fitness (in Chinese). Beijing, China: People's Sports Publishing House.

[ref60] Tucker P. (2008). The physical activity levels of preschool-aged children: a systematic review. Early Child. Res. Q. 23, 547–558. doi: 10.1016/j.ecresq.2008.08.005

[ref61] Wassenaar T. M. Wheatley C. M. Beale N. Salvan P. Meaney A., Possee J. B. Johansen-Berg H. (2019). Effects of a programme of vigorous physical activity during secondary school physical education on academic performance, fitness, cognition, mental health and the brain of adolescents (fit to study): study protocol for a cluster-randomised trial. Trials, 20::189. doi: 10.1186/s13063-019-3279-6, PMID: 30940164 PMC6444886

[ref62] Westfall D. R. Gejl A. K. Tarp J. Wedderkopp N. Kramer A. F. Hillman C. H.. (2018). Associations between aerobic fitness and cognitive control in adolescents. Front. Psychol. 9:1298. doi: 10.3389/fpsyg.2018.01298 30158882 PMC6104451

[ref63] Wexler B. E. Iseli M. Leon S. Zaggle W. Rush C. Goodman A.. (2016). Cognitive priming and cognitive training: immediate and far transfer to academic skills in children. Sci. Rep. 6, 1–9. doi: 10.1038/srep32859 27615029 PMC5018694

[ref64] WHO Multicentre Growth Reference Study Group de Onis M. (2006). WHO child growth standards based on length/height, weight and age. Acta Paediatr. 95, 76–85. doi: 10.1111/j.1651-2227.2006.tb02378.x 16817681

[ref65] Wick K. Kriemler S. Granacher U. (2021). Effects of a strength-dominated exercise program on physical fitness and cognitive performance in preschool children. J. Strength Cond. Res. 35, 983–990. doi: 10.1519/JSC.0000000000003942, PMID: 33752222

[ref66] Yoong S. L. Lum M. Jones J. Kerr E. Falkiner M. Delaney T.. (2020). A systematic review of interventions to improve the dietary intake, physical activity and weight status of children attending family day care services. Public Health Nutr. 23, 2211–2220. doi: 10.1017/S1368980019005275, PMID: 32383429 PMC10200613

[ref67] Zhexiao Z. Huanhuan M. Huanbin Z. Rui B. Yuanyuan L. (2016). An empirical study on promoting gross motor skill of preschoolers aged 5 to 6 through functional training. J. Chengdu Sport University 42, 16–22. doi: 10.15942/j.jcsu.2016.05.003

[ref68] Zhixiong Z. Shanshan D. Jun Y. Quan F. Hong R. Zenong Y. (2018). Improving physical fitness and cognitive functions in middle school students: study protocol for the Chinese childhood health, activity and motor performance study (Chinese CHAMPS). Int. J. Environ. Res. Public Health 15:976. doi: 10.3390/ijerph15050976, PMID: 29757933 PMC5982015

[ref69] Zhixiong Z. (2017). “Physical function training and physical fitness in middle school students” in Theory and practice of physical function training for middle school students (Beijing, China: People’s sports publish house of China).

